# A single workflow for multi-species blood transcriptomics

**DOI:** 10.1186/s12864-024-10208-2

**Published:** 2024-03-16

**Authors:** Elody Orcel, Hayat Hage, May Taha, Noémie Boucher, Emilie Chautard, Virginie Courtois, Adrien Saliou

**Affiliations:** 1 BIOASTER, 40 Avenue Tony Garnier, Lyon, 69007 France; 2 SANOFI, 1541 Av. Marcel Mérieux, Marcy-L’Étoile, 69280 France

**Keywords:** Preclinical models, Clinical models, Blood samples, RNA extraction, Library preparation, Total RNA sequencing, Transcriptomics, Data analysis, Quality controls, Standardization, Workflow, Report

## Abstract

**Background:**

Blood transcriptomic analysis is widely used to provide a detailed picture of a physiological state with potential outcomes for applications in diagnostics and monitoring of the immune response to vaccines. However, multi-species transcriptomic analysis is still a challenge from a technological point of view and a standardized workflow is urgently needed to allow interspecies comparisons.

**Results:**

Here, we propose a single and complete total RNA-Seq workflow to generate reliable transcriptomic data from blood samples from humans and from animals typically used in preclinical models. Blood samples from a maximum of six individuals and four different species (rabbit, non-human primate, mouse and human) were extracted and sequenced in triplicates. The workflow was evaluated using different wet-lab and dry-lab criteria, including RNA quality and quantity, the library molarity, the number of raw sequencing reads, the Phred-score quality, the GC content, the performance of ribosomal-RNA and globin depletion, the presence of residual DNA, the strandness, the percentage of coding genes, the number of genes expressed, and the presence of saturation plateau in rarefaction curves. We identified key criteria and their associated thresholds to be achieved for validating the transcriptomic workflow. In this study, we also generated an automated analysis of the transcriptomic data that streamlines the validation of the dataset generated.

**Conclusions:**

Our study has developed an end-to-end workflow that should improve the standardization and the inter-species comparison in blood transcriptomics studies. In the context of vaccines and drug development, RNA sequencing data from preclinical models can be directly compared with clinical data and used to identify potential biomarkers of value to monitor safety and efficacy.

**Supplementary Information:**

The online version contains supplementary material available at 10.1186/s12864-024-10208-2.

## Background

Advances in next generation sequencing (NGS) have revolutionized the analysis of transcriptome. Blood transcriptomics is widely used to identify gene expression signatures and potential biomarkers for diagnosis, prognosis, and monitoring the response to a vaccine or a treatment, environmental changes, or pathogenesis [1–4].

Although there are plenty of blood transcriptomics methods available to the scientific community, no clear consensus is available regarding the key points to be addressed for a robust transcriptomic workflow, from sample collection through to data analysis. To facilitate the implementation of these methods into preclinical and clinical routine practice, standardized methods are needed.

For a standardized analysis, the volume of blood collected may vary between different species [5, 6]. In small animals, the volume of blood that is practical to sample is often lower than in larger animals and humans, or lower in case of longitudinal studies due to repeated sampling [7, 8]. It is of importance to stabilize the collected blood as soon as possible [9–11], (i) to limit degradation of RNA, (ii) to minimize the risk of non-specific cell activation, and (iii) to allow for sample investigation several days or months later and/or at another analysis laboratory [12].

In the blood, the most abundant transcripts are ribosomal RNA (rRNA), which comprises 80% to 90% of total RNA, and globin mRNA, which comprises up to 80% of the protein-coding genes derived mRNA [13–15]. Both types of transcripts are often considered as non-informational. If not depleted, these abundant transcripts can interfere with or mask the measurement of the informational RNA, notably other mRNA types mainly found in a much lower proportion, around 5% of total RNA [15, 16]. Hence hybridisation capture of polyadenylated RNA (poly[A]) using oligo-dT probes is used to enrich mRNA from non-informational types. However, due to the presence of polyA tails, mRNA encoding globin is also captured. To mitigate this, hybridisation capture methods can be applied using globin-mRNA–targeting probes [17]. The drawback is that the design and the production of such species-specific probes, sometimes for infrequently considered species, are time consuming, not cost-effective, and may introduce bias into absolute transcript profiling.

Due to reductions in sequencing cost, total RNA sequencing is emerging as an alternative method in the analysis of blood transcriptome [18, 19]. In this approach, all the RNAs present in the sample, including mRNA, are sequenced, after non-informational RNA (rRNA and globin mRNA) has been removed during library preparation. It provides a comprehensive view of the blood transcriptome and is commonly used to identify and quantify the expression levels of all genes in a sample, as well as to identify novel transcripts and splice variants. Remarkable studies have shown that a total RNA library is able to capture a significantly higher number of protein coding mRNAs than the mRNA-Seq, as not all mRNAs necessarily contain a poly(A) tail at their 3’ ends [20, 21]. This approach also works well for degraded RNAs, in which poly(A) tails may have been lost, reducing a potential bias in transcript identification and quantification.

Commercial solutions are mainly available to deplete rRNA and globin mRNA for frequently considered species, including human, mouse and rat [22]. For other species, blood transcriptomics methods are not well established, thus limiting the emergence of studies from those species. Based on an innovative probe-free depletion strategy, the Zymo-Seq RiboFree Total RNA Library Preparation kit (Zymo Research) has the potential to be used on any species but little is known regarding its application for animals used in preclinical models [23]. Hence, a well-defined blood transcriptomics workflow [24] that can potentially be applied to any animal species would help minimize the variability and ensure the reliability and reproducibility of the results.

In this study, we describe a workflow that allows generating reliable transcriptomic data from the whole blood total RNA of any species, starting from sample collection through to data analysis. This streamlined workflow includes the choice of the RNA extraction protocol, the preparation of sequencing libraries and their validation, followed by Illumina sequencing. In our study, the Zymo-Seq protocol was tested in four different species: mouse, rabbit, non-human primate (NHP, *Macaca fascicularis*), and human. A particular attention was paid to the development of a bioinformatics pipeline focused on quality assessment, which can generate a useful report for the visualization of quality controls. In our study, we focused on total RNA sequencing and summarized the key criteria that need to be considered in the workflow, with their associated thresholds, that should guarantee reliable RNA sequencing data for multi-species comparisons.

## Methods

### Collection of blood samples

Blood samples were collected in triplicates from six rabbits, six mice, six human donors and four NHP. Blood was collected into PAXgene tubes (#BD762165, BD Biosciences) for mouse, NHP and human samples or into lithium heparin tubes for the rabbit samples (#13,526,530, Greiner). For PAXgene conservation, a blood:reagent ratio of 2.5:6.9 was conserved, regardless the species [14–17]. Next, 150 µl (mouse), 250 µl (NHP) and 2.5 ml (human) of each blood sample was dispensed into 1.5 ml Eppendorf tubes or 15 ml Falcon tubes to which 414 µl, 690 µl and 6.9 ml PAXgene reagent were added, respectively. For the rabbit samples, 3 ml of total blood were dispensed into 3 ml lithium-heparin tubes (#13,526,530, Greiner). 1 ml of heparinized blood was then mixed with 2.8 ml of PAXgene reagent into 15 ml Falcon tube (#352,097, Falcon). After collection, the tubes were inverted 10 times and stored upright at room temperature (18 °C–25ºC) for a minimum of 2 h and a maximum of 72 h before transferring to a freezer at -20 °C for 24 h and then at -80 °C until RNA extraction.

### RNA isolation

Total RNA was extracted using the Maxwell HT simplyRNA kit, Custom (#AX2420, Promega), treated with DNase I and eluted in 50 µl of nuclease-free water. Extracted RNA was purified using the RNA Clean and Concentrator-5 kit (#R1013, ver.2.2.1, Zymo Research), including the step of DNase I treatment (5U/µl of DNase I, 15 min at 25 °C) at the beginning of the protocol. Before implementing this second DNase I treatment for all the samples, we compared the sequencing data obtained from RNA rabbit samples treated once or twice with DNase I (See Results and Fig. 9).

Note that RNA extracted from the mouse blood samples was not submitted to this second DNase I treatment, as the blood volume and consequently the RNA yield were too low. Total RNA quality and quantity were assessed using the Fragment Analyzer Standard RNA (15nt) Kit (#DNF-471–0500, Agilent) on the Fragment Analyzer system (Agilent). For the mouse samples, quality control of RNA was assessed using the Agilent RNA 6000 Pico Kit (#5067–1513, Agilent) on the Bioanalyzer system (Agilent).

### RNA library preparation

A total RNA library was prepared using the Zymo-Seq RiboFree Total RNA Library Kit (#R3003, Ver.1.04, Zymo Research) with modifications. An input of 250 ng of total RNA was used for the library preparation from human and rabbit samples. An input of 100 ng and 50 ng was used for NHP and mouse samples, respectively. The depletion step of non-informational transcripts was conducted for 4.5 h for all species. The number of PCR cycles was adjusted according to the input of total RNA and the manufacturer’s recommendations: 15 cycles were used for human and rabbit samples and 16 cycles for mouse and NHP samples. The libraries were double-purified using the Select-a-Size MagBead Concentrate (Zymo Research) at the 0.9X bead:library ratio and eluted in 15 µl of nuclease-free water. Ready-to-sequence libraries were quantified using the QuantiFluor One dsDNA kit (# E4870, Promega) on the GloMax system (Promega). Quality control was performed using the High Sensitivity NGS Fragment Analysis Kit (#DNF-474, Agilent), on the Fragment Analyzer system (Agilent). Prior to sequencing, the samples of each species were randomized into pools of four samples. The sequencing of the four samples onto the same flow cell was made possible as one unique barcode was added per sample during the library preparation. Randomization is carried out on several criteria allowing a homogeneous distribution of samples between runs (e.g.: group, time-point).

### Sequencing

Sequencing was performed on a NextSeq500 system (Illumina) using the NextSeq 500/550 High Output Kit v2.5 (150 Cycles) in a 2 × 75 bp mode, in accordance with Illumina’s recommendations.

### Data preprocessing

After sequencing, BCL files were demultiplexed into separate FASTQ files for each sample, using bcl2fastq tool v2.20.0.422. The sequencing quality control was checked using FASTQC v.0.11.5 [25]. FASTP v.0.20.1 [26] was then used to remove low-quality reads and to trim any Illumina adapters. Sortmerna v3.0.3 [27] was used to identify residual rRNA reads remaining after the depletion process, by aligning the reads against rfam and SILVA rRNA reference database [28]. To estimate the percentage of remaining globin sequences, the reads were aligned against a reference index of globin genes. Globin genes were identified from the user-specified GTF annotated files of the species of interest and their corresponding sequences were used to build the reference index. Filtered reads were then aligned to the corresponding reference genome or transcriptome using STAR v.2.7.9a [29]. GRCh38.p14, MFA1912RKSv2 GRCm39, OryCun2.0 NCBI assemblies were used for human, NHP, mouse and rabbit, respectively. RSeQC package v.3.0.0 [30] was used to assess the mapped reads distribution, coverage uniformity and strand specificity. SeQmonk v1.48.1 was used to deeply visualize the distribution of mapped reads against the annotated genome. The aligned reads were used to quantify the number of reads from each genomic feature and to generate the count-expression matrix for each gene in each sample, using Salmon v.0.12.0 [31]. To reduce the impact of genes considered as background noise, some filtering and normalization methods were used. A gene was considered as background noise and filtered out if it has fewer than 10 counts across all samples. After filtering, the count expression matrix was normalized to eliminate technical variability using Relative Log Expression (RLE) from DESeq2 v. 1.36 [32]. After the count expression matrix was normalized, the number of protein coding genes was computed for each sample. Rarefaction curves were computed using R v.4.2.1. A single graphical report per analysis, that includes all the quality control plots across samples, was generated using multiQC v.1.12 [33]. The different steps were connected using the workflow management system Snakemake and the package management system Conda.

## Results

### Study design

**Fig. 1:**
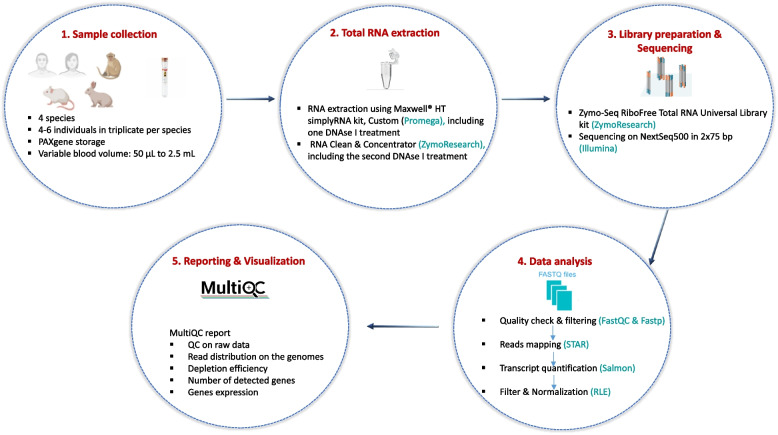
Blood transcriptomics workflow from sample collection to data report. (1) Blood samples from all species (human and model animals) were collected on PAXgene tubes. The PAXgene buffer stabilized samples before extraction. After the first step-by-step freezing, samples were frozen at -80 °C until extraction. Samples came from 6 donors for human, rabbit, and mouse, and 4 donors for NHP in triplicate. (2) Total RNA was manually extracted with Maxwell HT Simply RNA kit custom (#AX2420, Promega). RNA was then processed using the RNA Clean and Concentrator kit, including an additional DNase-I treatment (#R1013, ver.2.2.1, Zymo Research). (3) Total RNA libraries were prepared using the Zymo-SeqRiboFree Total RNA Library Kit (#R3003, Ver.1.04, Zymo Research) which integrates the depletion of globin mRNA and rRNA. The conditions of the library preparation were adjusted according to the associated species and the extraction yields. Libraries were sequenced on the NextSeq500 system (Illumina). (4) Quality control of the data was performed using an in-house pipeline that includes four main stages: (i) quality assessment, (ii) read mapping, (iii) transcript quantification and (iv) filtering and normalization. (5) The pipeline generated a final report assembling all the necessary plots to evaluate the quality of the data

Blood samples were collected from individuals of four different species including human, mouse, NHP, and rabbit. Samples from a total of 22 individuals (4 NHPs, 6 human donors, 6 mice, and 6 rabbits) were analysed in triplicates using a single total RNA-Seq workflow from sample collection to data analysis (Fig. 1). To guarantee the quality of samples for long-term storage, blood was collected in PAXgene tubes (See Methods for a detailed description). RNA was extracted using the Maxwell HT simplyRNA kit, Custom (Promega). To ensure complete DNA removal, RNA samples were subsequently purified and processed with a second round of DNase I, except for the mouse samples. Total RNA-Seq libraries were prepared using the Zymo-Seq RiboFree Total RNA Library Kit (Zymo Research). Some species-specific adaptations were implemented (See Methods for a detailed description and Table 1). Libraries were sequenced using the Illumina technology. Following sequencing, the performance of the transcriptomic workflow was evaluated using a dedicated pipeline that included four main stages: (i) quality verification, (ii) read mapping, (iii) transcript quantification and (iv) filtering and normalization. These evaluations were captured in a multi-QC report, that enabled the rapid validation of the sequencing data quality before downstream analysis. In this study, three mouse samples (Mouse3_2, Mouse5_1 and Mouse6_2) were removed from the analysis due to a low extraction yield and one rabbit sample (Rabbit4_2) was lost at the collection step, resulting in a total of 62 samples being successfully processed and analysed.

**Table 1:** Experimental conditions for each species, including the blood volume, the use of the additional DNase I treatment, total RNA input, the time of depletion and the number of PCR cycles

Species	Blood Volume (mL)	Two DNase I treatments	Total RNA input (ng)	Time depletion (h)	Number of PCR cycles
Human	2.5	Yes	250	4.5	15
NHP	0.25	Yes	100	4.5	16
Rabbit	1	Yes	250	4.5	15
Mouse	0.15	No	50	4.5	16

### Quality and quantity of extracted RNA

When extracting RNA from blood samples, RNA ought to be well-preserved and in a large enough quantity to prepare the transcriptomics libraries. As the volume of blood that can be collected in preclinical models is often limited, the chosen protocol needs to perform well on a large range of blood volumes from few µl to several ml. The protocol also needs to be compatible with different stabilizing solutions as the routine practice can be different. In the present study, we evaluated the performance of the Maxwell HT simplyRNA kit (Promega) in terms of RNA yield and RNA quality. A protocol that performs poorly on these criteria may likely skew measured transcripts compositions, as only a small -and most likely non-representative- portion of transcripts present in the original sample would be analysed.

**Fig. 2:**
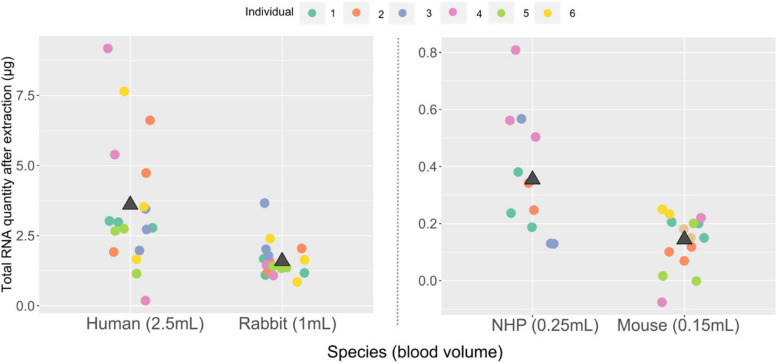
The quantity of extracted RNA from blood samples (µg). Each colour represents an individual. The average quantity between samples per species is shown as a black triangle. The original blood volume is shown in parentheses

**Fig. 3:**
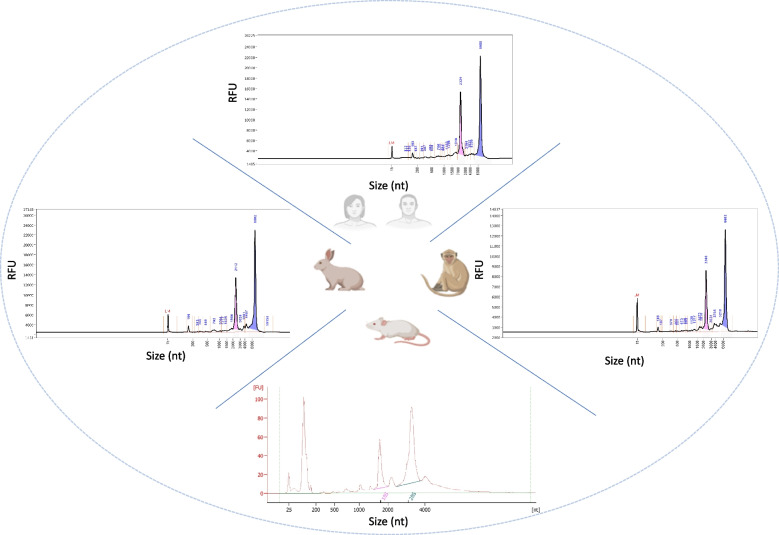
Total RNA profiles after extraction. One example of extracted RNA profiles is shown per species (Samples: Human1_3, NHP2_1, Mouse4_2 and Rabbit3_2). The profiles were generated using the Bioanalyzer system for the mouse samples and using the Fragment Analyzer for the three remaining species

The extraction yield showed considerable variations between the four species (Fig. 2). Mean yield (± SD) was 3.6 µg (± 2.3) and 350 ng (± 0.16) for human and NHP respectively, with a minimum of 100 ng for each species. For mouse, the mean yield was 130 ng (± 0.07). Less than 50 ng were recovered for two samples from three different mice (40 and 20 ng). They were thus excluded from downstream analysis as the 100 ng minimum input for library preparation, as recommended by Zymo Research, was not attained. For rabbit, the mean yield was 1.6 µg (± 0.63) with a minimum of 900 ng. The extraction yield was mainly dependent on the initial blood sample volume. Extracted RNA quantities increased with the collected blood volume regardless of the storage methods (i.e., lithium-heparin tubes for rabbit samples and PAXgene tubes for samples of other species). Among replicates, extraction yield variability was highest in humans, mostly due to the overall higher yield compared to other species (Supplementary Table 1). Regarding the RNA quality, the mean (± SD) RNA-integrity-number (RIN) values were 8.8 (± 0.3) for human, 9.9 (± 0.08) for NHP, 9.6 (± 0.2) for mouse, and 9.6 (± 0.2) for rabbit samples, confirming the good performance of the extraction protocol (Supplementary Table 1). Figure 3 shows the gel electrophoresis profiles of RNA extracted for three individuals from each of the four species.

### Library preparation and sequencing

**Fig. 4:**
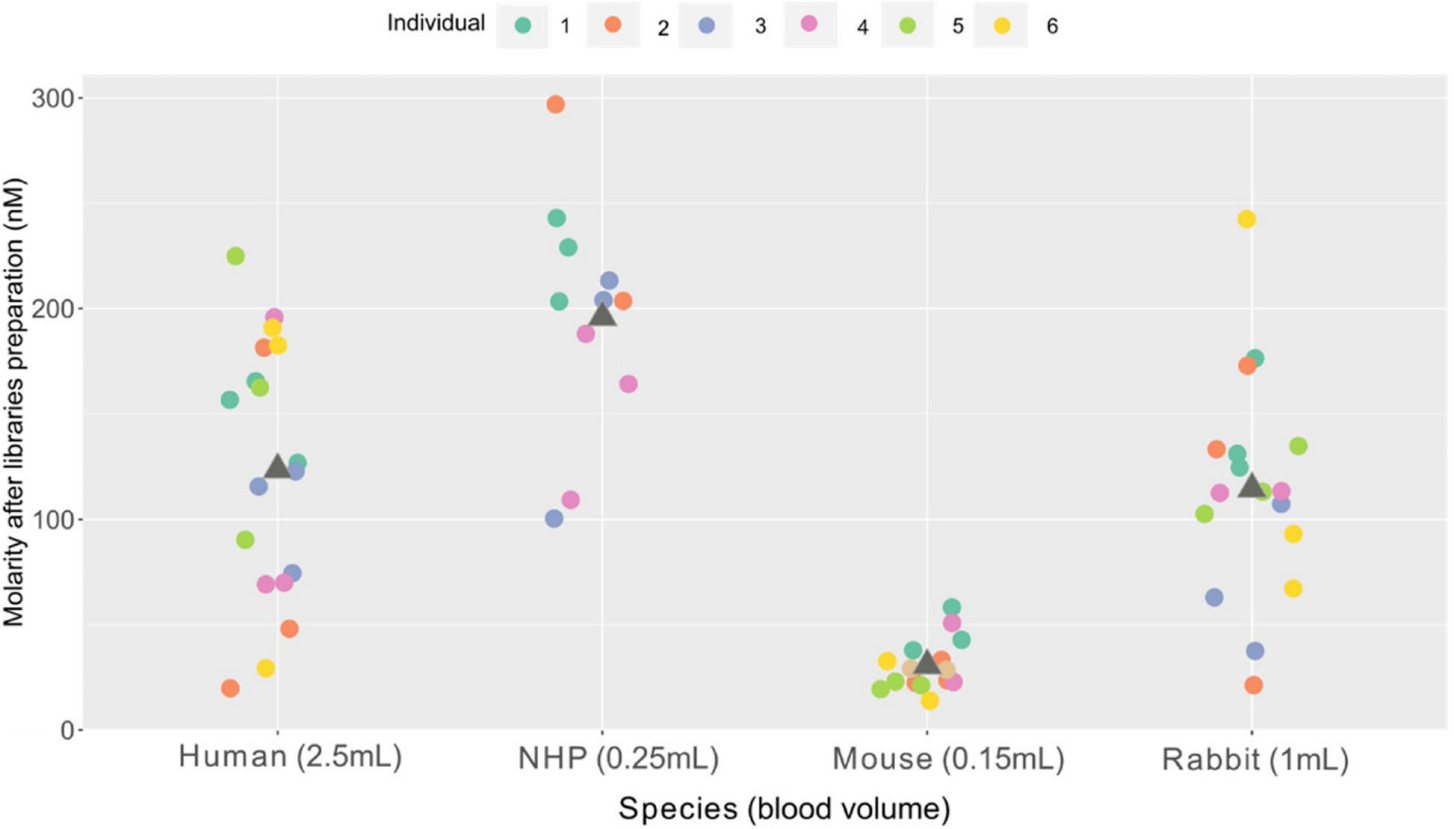
Quantity of the libraries (nM). The library molarity was calculated from the concentration (ng/µl) and the average size. Each colour represents an individual. The average quantity between samples per species is shown as a grey triangle. The original blood volume is shown in parentheses

In our study, the preparation of the library involved taking into consideration: (i) the multi-species origin of the RNA, (ii) the variability in the RNA extraction yield, and (iii) the requirement to deplete both rRNA and globin. We evaluated the performance of a commercially available solution for RNA sequencing library preparation including a probe-free rRNA depletion module (Fig. 4) with an RNA input for library preparation based on the minimum of RNA recovered for each species. An input of 250 ng of total RNA was used for human and rabbit samples. By contrast, an input of 100 ng was used for NHP samples, as the minimum recovered RNA was around 170 ng. Due to the limited volume of blood, the RNA input was only 50 ng for the mouse samples, which was below the minimal recommended input at the time of the study (100 ng). All the libraries were successfully prepared, regardless of the species and of the amount of RNA used (Fig. 4). As expected, the library molarity was higher when more RNA was used as input. For human, library molarity ranged from 19 to 224 nM with a mean of 123 nM. For NHP, library molarity was between 100 and 297 nM with a mean of 191 nM. For rabbit, the molarity ranged from 21 to 242 nM with a mean of 114 nM. For mouse, the library molarity was between 19 and 58 nM, which was sufficient for library loading onto the sequencing flow-cell.

In addition, the library fragment size was very similar between species, regardless of the quantity of RNA used as input (Supplementary Table 2). We obtained homogeneous profiles following a Gaussian distribution with a mean fragment size of 350 bp, which was in good agreement with the supplier’s indications. No small fragments (inferior to 200 bp), which are often attributed to primer dimers, or larger fragments that have been considered as residual genomic DNA, were observed by gel electrophoresis.

After sequencing, the number of reads generated per sample was consistent across all samples and species, which is usually desirable to ensure data comparison (Supplementary Table 2). We obtained an average of 122 ± 32 × 10^6^, 125 ± 11 × 10^6^, 110 ± 11 × 10^6^, 122 ± 26 × 10^6^ reads for human, NHP, mouse, and rabbit samples, respectively.

### Quality controls of raw data

After sequencing, the data quality was assessed by dry-lab metrics. First, the Phred score, which is a base quality score that estimates the probability that each base call is correct, showed a base call accuracy of over 99.9% for all the samples (Supplementary Figure 1). On average, 96% of the total raw reads of each sample were considered as of high quality and were used for the downstream analysis. Total RNA-Seq data may suffer from the presence of foreign species RNA, which may highly affect the downstream analysis. In our study, the presence of multi-species RNA was assessed based on the GC content ratio and the percentage of the genome mapping. Although it is difficult to identify the species from GC-content readout, a shift from the expected GC-content ratio or multiple GC peaks may suggest the presence of contamination. Based on NCBI reference genomes, we defined a species theoretical GC-content ratio of 40.4%, 40.5%, 41.5%, and 43.5% for human, NHP, mouse, and rabbit, respectively. Here, the four species studied showed clear peaks around the expected GC content ratios (Supplementary Figure 2). However, in some mouse samples, an additional smaller peak was observed, which may have been associated with globin reads. The absence of contamination was further supported by the observation that < 10% of reads failed to align to the corresponding reference genome (Supplementary Figure 3).

### Performance of rRNA and globin depletion

**Fig. 5:**
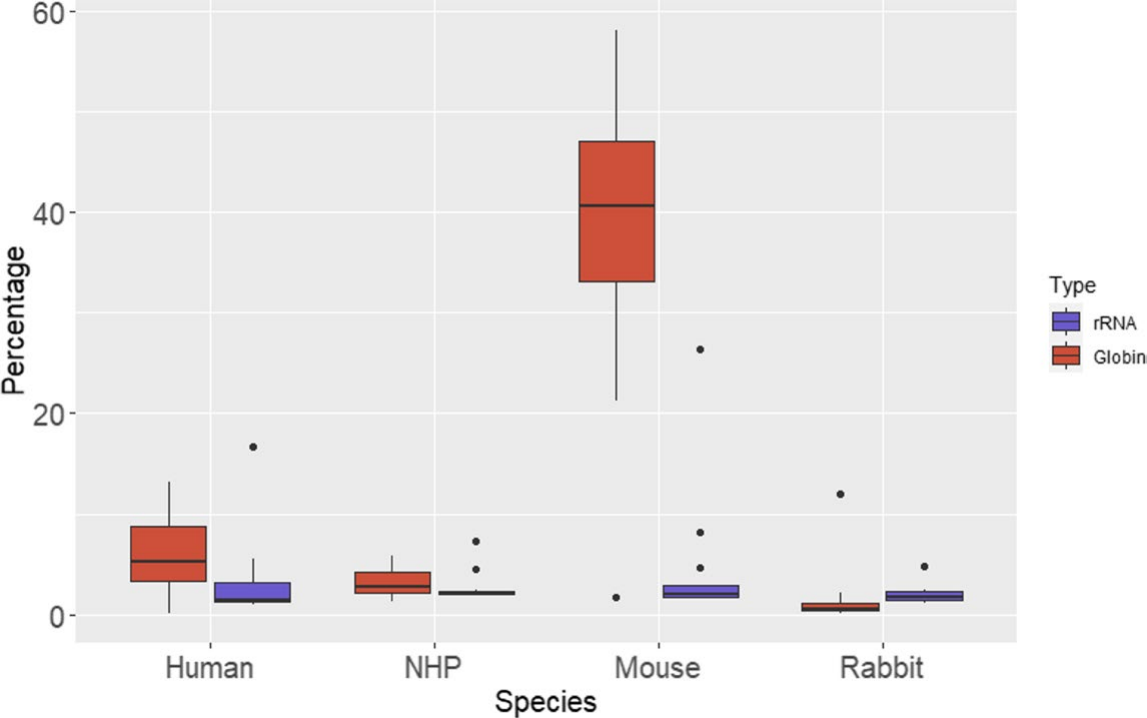
Performance of rRNA and globin depletions. Percentage of reads aligning to the rRNA reference database (blue) and to the globin index reference (red) for each of the four species

In total RNA-Seq experiments from blood, it is essential to remove as much globin transcripts and rRNA as possible to reduce the sequencing cost. In this study, after the depletion step, we estimated the amount of globin transcripts and rRNA in each sample by aligning the reads to the rRNA reference database and globin mRNA reference indexes. An efficient rRNA depletion was observed for all four animal species as < 10% of reads were identified as rRNA (Fig. 5). An efficient globin mRNA depletion (ie, < 10% of reads) was also observed for all species except the mouse, for which > 40% reads were globin mRNA. The main reason for this difference in globin mRNA depletion is likely due to the RNA quantity used. Indeed, while at least 100 ng of RNA was used for human, NHP, and rabbit samples, only 50 ng was used for mouse samples. When the amount of mouse RNA was increased to 150 ng, < 5% of reads were globin mRNA after depletion (Data not shown). Consequently, our results strongly suggest that the efficiency of depletion may be related to the initial quantity of RNA used for library preparation.

### Read distribution over genome features

**Fig. 6:**
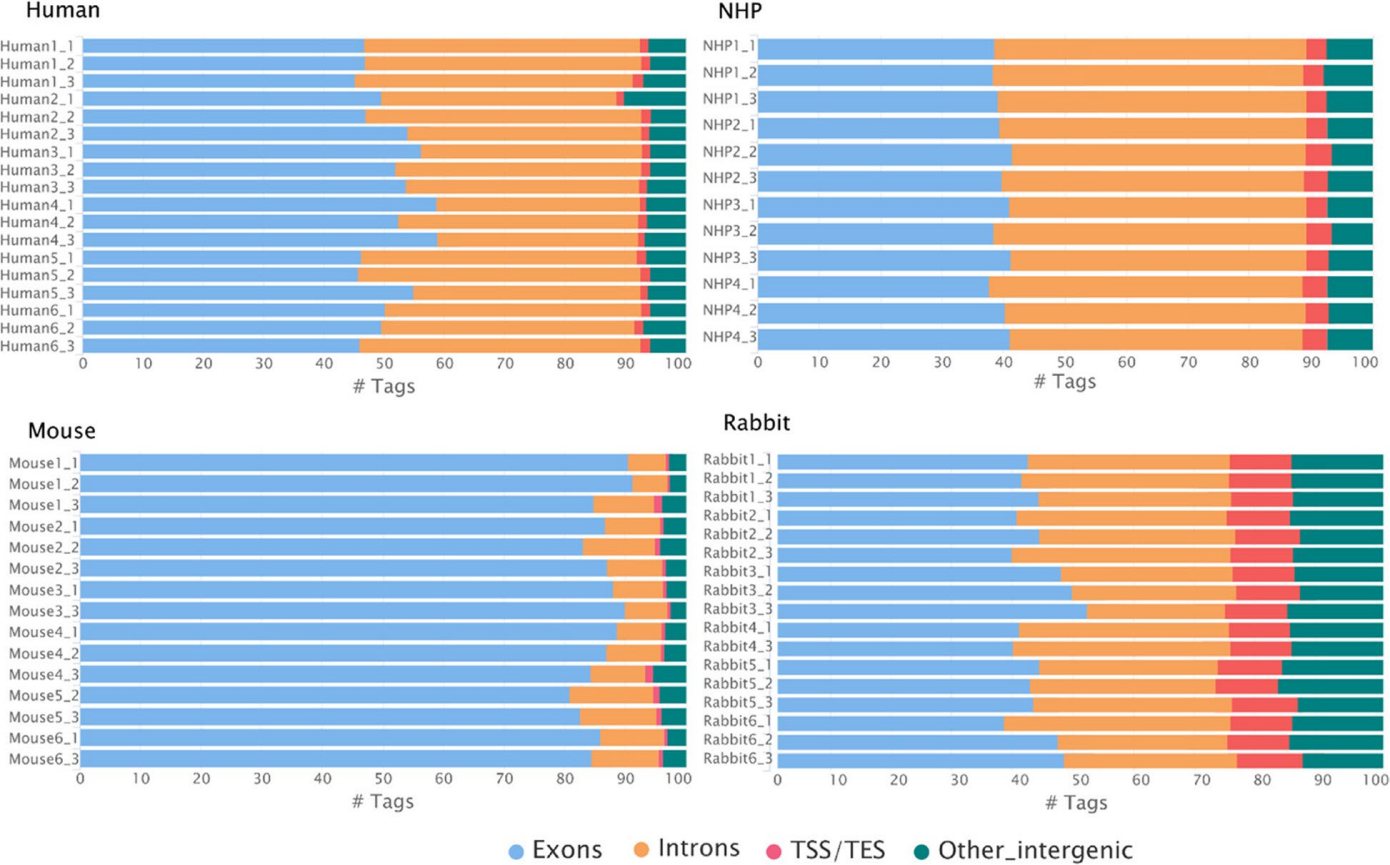
Read distribution by genomic features. Bar plots show the percentage of reads mapping over the different genome features for each species: in blue, the exons, in orange, the introns, in red, the TSS/TES (Transcription start and end sites), and in green, other intergenic regions which regroup reads mapping outside the genes on the genome

We further examined how consistent the read distribution was over the genome features, including exons, introns, and intergenic regions (Fig. 6). In total RNA-Seq, it is expected to find a significant proportion of reads mapping to introns and intergenic regions, in contrast to a polyA RNA method. A low variability was observed in the genomic features distribution among samples and technical replicates of each species, indicating that the workflow consistently produces reliable results. Percentages of exonic RNAs ranged from 45 to 58% in humans, 36 to 42% in NHP, 80 to 91% in mice, and 35 to 50% in rabbit. Regarding the intergenic reads, their proportions were relatively low in human, NHP, and mouse samples (5% on average), and slightly higher in rabbit samples (15% on average).

Based on NCBI annotations of protein coding genes, we investigated read distribution over gene biotypes, including miscellaneous RNA (misc_RNA), long non-coding RNA (lncRNA) and mitochondrial rRNAs (Mt_rRNA) (Supplementary Fig. 4). The percentages of reads assigned to these four biotypes were relatively uniform among species, with protein coding genes representing over 70% of the total reads. Inter-species differences in the distribution of biotypes tended to be small. One such difference was a higher proportion of Mt_rRNA reads in rabbit samples (25%) compared to those of other species (7–15%). Also, there was a higher proportion of lncRNA in human samples (7%) while these were almost absent in other species (0–2%), possibly due to higher annotation rates [34].

### Number of expressed genes

**Fig. 7:**
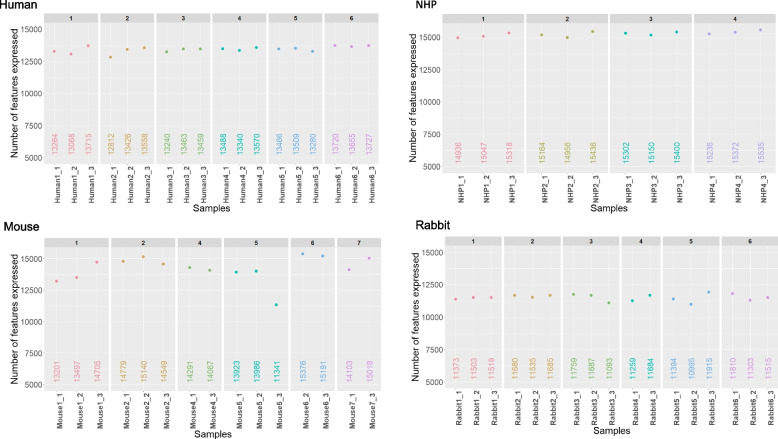
Number of detected genes. The number of genes detected was computed as expressed with at least 10 reads. Each dot represents an individual and each colour samples from the same triplicate

We next evaluated the number of detected genes that have more than ten mapped reads (Fig. 7). The average number of detected genes can vary widely depending on several factors, including the studied species, the degree of non-informational RNA depletion, and the sequencing depth. We obtained a homogeneous number of expressed protein-coding genes among samples from a same species, with an average of 13 464, 13 605, 11 527 and 11 150 genes, in human, NHP, mouse, and rabbit samples, respectively. The intra-species variability in the number of coding genes was low, suggesting that the complete workflow can generate consistent results. Although intra-species differences appeared to be related to the number of reads, it is worthwhile to note that the relatively minor difference in the number of genes identified from the two most divergent samples (915 genes in human) was associated with an almost threefold increase in the number of reads (i.e., from 73 × 10^6^ to 201 × 10^6^).

### Transcriptomics report

**Fig. 8:**
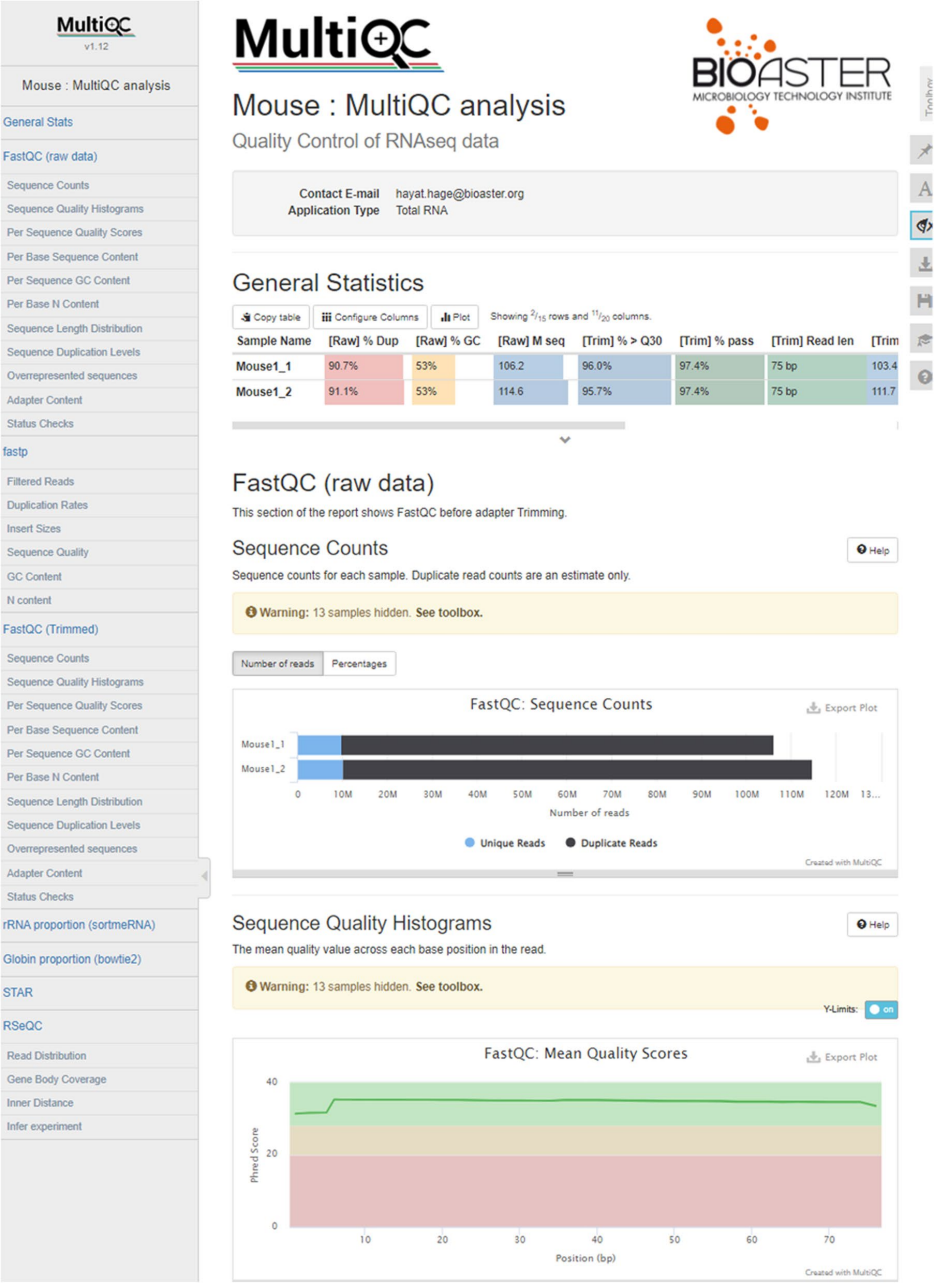
Example of RNASEQ-QC analysis for transcriptomics QC

Based on already available open-source tools, we developed an in-house pipeline, called RNASEQ-QC [35], for conducting quality controls on transcriptomic data (Fig. 8). RNASEQ-QC was compared to the RNA-Seq nf-core pipeline to further validate the reliability of our workflow (Supplementary Fig. 6). RNASEQ-QC allows the user to quickly generate a comprehensive report assembling all the graphs and tables required for quality assessment. The pipeline generates an interactive MultiQC report incorporating graphs with dynamic sample-filtering features. Additional QC graphs generated in R were appended to the end of the MultiQC report to provide a comprehensive overview of all the dry-lab QC metrics discussed in this paper.

### Detection of residual genomic DNA

**Fig. 9:**
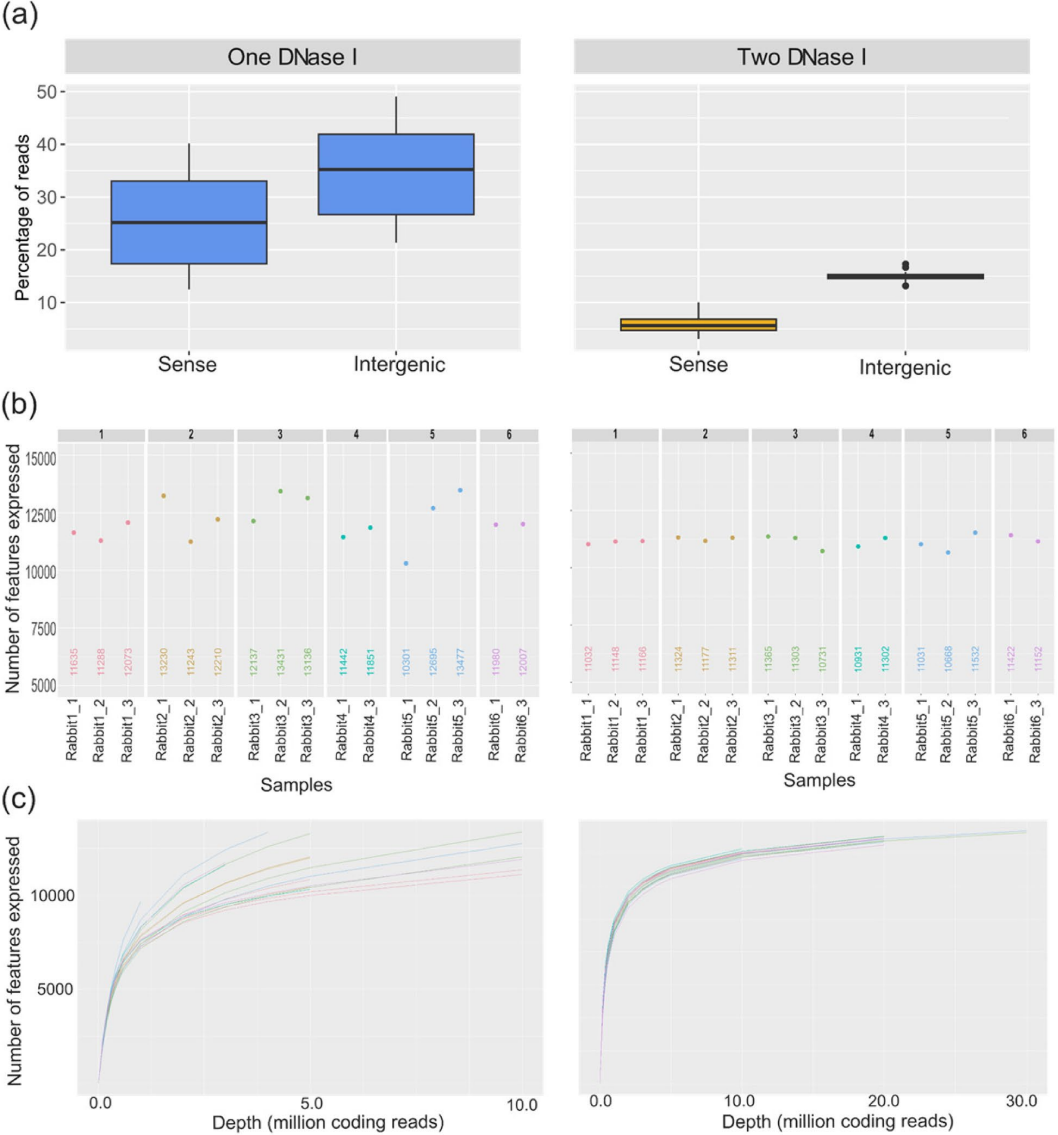
Four QCs to detect residual genomic DNA in rabbit samples. The samples were analysed after a single DNase I treatment or double DNase I treatment. **a** Boxplots show the effect of DNase I treatment on read directionality (percentage of sense reads and the percentage of intergenic reads). **b** Dot plots describe the number of expressed genes detected. **c** Rarefaction curves describe the relationship between the sequencing depth and the number of detected genes, with each curve corresponding to one sample

One major bias encountered in total RNA-Seq may arise from the presence of residual genomic DNA as the RNA extraction protocol may also extract DNA traces. For this reason, one or two rounds of DNase I treatment can be used to deplete residual genomic DNA after RNA extraction, depending on the type of sample. In practice, residual genomic DNA can be visualized on an electrophoresis gel. It often appears as a smear between RNA bands or can be identified as large fragments. However, common wet-lab criteria may not be sufficient to identify residual genomic DNA from an RNA profile erroneously appearing as genomic DNA-free. If not properly identified, the presence of residual genomic DNA may significantly impact the accuracy of quantitative data [36]. During the set-up experiments, we compared the data generated from rabbit RNA after a single or after two consecutive DNase I treatments. The samples were evaluated using several dry-lab criteria, including the strandness of the library, the percentage of intronic reads, the number of genes detected, and the rarefaction curve (Fig. 9). Because DNA is double-stranded and does not have a directionality bias, DNA-derived reads will not have a clear orientation relative to the reference genome. This lack of directionality makes DNA presence easy to spot in stranded protocols of RNA-seq libraries. For single DNase I-treated RNA, we could assign a high proportion of reads to the sense strand (> 20% on average and reaching 40% in some samples), which was not expected as the Zymo-Seq solution does not generate sense stranded libraries (Fig. 9a). When applying two consecutive DNase I treatments, this proportion was < 10% for all the samples.

To confirm the presence of residual genomic DNA for single DNase I-treated RNA, we then estimated the percentage of reads mapping on intergenic regions (green, Fig. 9a). A high proportion of intergenic-assigned reads was recently shown to be related to the presence of residual genomic DNA. When RNA was treated twice with DNase I, the percentage of intergenic regions mapped was < 30%. This observation was confirmed by the mapping of reads against the rabbit reference genome. In single DNase I-treated RNA, the presence of DNA appeared as a constant background of reads aligning throughout the genome, and not affected by gene boundaries or by directionality (Supplementary Fig. 5). When RNA was treated twice with DNase I, the reads mostly mapped to the genes. As the next step of our assessment of the two conditions, we then estimated the number of genes detected (Fig. 9b). In single DNase I-treated samples, the number of expressed genes tended to be heterogeneous and ranged between 10 000 and 13 000 genes. This might be due to the nature of DNA-derived reads mapping to non-expressed genes, leading to their erroneous expression. By contrast, the variability in the number of expressed genes was low between replicates of the same sample or in samples subjected to double DNase I treatment. Next, rarefaction curves were computed from all samples subjected to either a single or double DNase I treatment, which allows to determine if the sequencing depth is sufficient to capture all the information. Interestingly, the plateau was not reached for most RNA samples subjected to the single DNase I treatment in contrast to the corresponding samples subjected to double DNase I treatment (Fig. 9c, Supplementary Fig. 7). Altogether, even if the wet-lab criteria suggested its absence, the above dry-lab criteria clearly indicated the presence of residual DNA contamination after single DNase I treatment, and its absence after double DNase I treatment. Based on these preliminary observations, all RNA samples were submitted to double DNAse I treatment before library preparation, except for the mouse samples for which the initial amount was too low to undergo this additional step.

## Discussion

Recent studies describing the implementation of blood transcriptomics workflows are mostly limited to a single species [37, 38]. Consequently, the performance of those workflows may not be guaranteed for other species of interest.

Our study is the first, to our knowledge, to evaluate a single blood transcriptomics workflow, from sample collection through to data analysis, that is suitable for both human samples and samples from three animal species commonly used in preclinical studies. This evaluation was carried out on 4 to 6 individuals of each species, with samples collected in triplicate, providing a total of 66 blood samples. The performance of the workflow was evaluated using various wet-lab and dry-lab criteria. To streamline the quality control of the data generated, we also developed a transcriptomics pipeline that summarises the different dry-lab criteria.

When a high number of samples need to be sequenced and this number exceeds the capacity of a single sequencing flow cell, the randomization of samples is required to limit batch effects that could introduce confounding effects from other biological factors and mask an underlying biological signal [39]. In this study, replicates of the same subject were randomized into different sequencing batches, to limit the bias.

Unlike human blood, there is no clear guidelines on collecting blood from preclinical animal models. When available, the impact of the chosen storage method is not well demonstrated in terms of RNA quality and quantity. In our study, we adapted the PAXgene solution [40], commonly used for collecting 2.5 ml of human blood samples, to smaller blood volumes collected from preclinical samples. To ensure some homogeneity between samples, regardless of the species, we adjusted the volume of PAXgene buffer to the collected blood volume, with respect to a constant blood:reagent ratio of 0.36. The PAXgene collection method represents the best solution for long-term freezing of RNA without affecting the stability of gene expression profiles, as described by Debey-Pascher et al. 2011 [9].

After blood collection, RNA was manually extracted using a commercially available kit [41]. This method enabled the isolation of good quality RNA for all samples evaluated (RIN > 8). The quantity of RNA extracted was proportional to the volume of blood collected. However, for a given species, we observed high variability between samples and replicates. We hypothesized that the method used to collect the blood may have a strong impact on the RNA yield due to laboratory practices, the intervention of different operators or the multiple and successive handling procedures of each sample [42].

Although total RNA-seq may require more sequencing depth compared to the standard mRNA-seq approach [43] leading to higher costs, it also offers a number of advantages such as successful library preparation from degraded and weakly concentrated RNA samples [44]. In this study, all libraries were successfully prepared for Illumina sequencing even with RNA inputs below the recommended 100 ng. The tested method has the advantages of (i) incorporating a probe-free depletion of both rRNA and globin mRNA during library preparation, (ii) avoiding the need for species-specific library preparations, and (iii) avoiding the use of generic depletion methods that might preferentially work on a given species [45]. By following our method, the need for species-specific reagents and probes, which commercial solutions often lack of, is overcome [22].

Remarkably, in this study, we were able to generate mouse libraries from as low as 50 ng by increasing the depletion time and the number of PCR cycles. Even if the rRNA depletion was satisfactory (< 10%), this led to suboptimal globin RNA depletion (50%), which was compensated by a high sequencing depth. However, after the completion of this study, Zymo Research released a new version of the protocol in which libraries can be prepared with as low as 10 ng, or with 1 ng with some modifications, including the increase of the depletion time at 4 h, and the increase of the number of PCR cycles to 14 or 15. These new recommendations are in line with the modifications we implemented during our study. Additionally, this approach may be suitable for sequencing degraded RNA with RIN < 8. A complementary study, not presented in this paper, was successfully carried out on a larger number of rabbit RNA samples, for which the RNA was degraded by several freeze–thaw cycles to give RIN scores between 5.5 and 7.

In addition to wet-lab criteria, a combination of various quality metrics must be considered to assess the quality of sample [17]. This includes the sequencing Phred score, the GC content, the efficiency of the depletion of unwanted blood transcripts, the read distribution over the genome features, the absence of contamination with foreign species, the presence of residual genomic DNA, and the number of expressed coding genes. In our study, the overall Phred score quality was excellent. The GC content confirmed the absence of contamination as the observed ratios matched the theoretical ones derived from the literature. The degree of depletion of unwanted transcripts was good overall when at least 100 ng RNA was used for library preparation, with < 10% of reads corresponding to rRNA and globin mRNA.

The distribution of the reads was found to be consistent, both between individuals and between replicates, for a given species. As expected, we observed differences in the distribution of genome features across the different animal species, with highest similarities found between human and NHP. This observation was in line with a previous study comparing humans and chimpanzees [46]. In addition to the quality of the available genome annotations, feature (biotype) distribution may also be impacted by factors such as the source tissue and the types of RNA sequenced, primarily poly-A RNA versus total RNA [47, 48]. Hence, intronic sequences appeared more frequently than would occur in poly-A selected RNA samples. Moreover, for a given species, a homogeneous number of expressed genes was observed, and a plateau was reached for the rarefaction curves for all samples. Altogether, the between-species and within-species characterisation of sequences suggested that our workflow generated consistent results.

As each dry-lab metric provides information about a particular aspect of the data, a failure in one metric does not necessarily exclude the sample from the study. In some circumstances, the overall quality can still be satisfactory, with little impact on the analysis, even if technical factors or variations in experimental protocols caused a specific metric to fail. It is also important to consider the consistency of quality assessments across samples that were processed at the same time, as this represents another key factor for a successful downstream analysis. This aspect was taken into account in designing the RNASEQ-QC pipeline that provided a rapid and global view of all the metrics for all study samples.

Unlike standard wet-lab QC such as Bioanalyzer or Fragment analyser profiles, the RNASEQ-QC pipeline was crucial for detecting the presence of residual genomic DNA. We identified different dry-lab criteria that signal abnormalities and heterogeneity between samples. This includes the proportion of sense vs antisense reads, the read distribution over the genomics features, the number of detected genes, the profiles of the rarefaction curves and the mapping of the sequencing reads against the genome. Our investigation suggested that a single DNase I treatment after RNA extraction was not sufficient to digest all DNA contained within a sample. The implementation of an additional DNase I treatment enabled the generation of robust data for the rabbit samples and could be generalized to the other species evaluated in this study.

Based on our work, there are some key criteria that could help establish a reliable end-to-end workflow (Table 2). These key criteria should reduce the time and cost of new transcriptomics studies and help comparing data generated in different studies. First, we recommend preparing the libraries with a minimum RNA input of 100 ng. This minimum ensures the efficient depletion of both rRNA and globin, leading to an expected decrease of their percentages below 10% and 30%, respectively. As described by Shin and al [49], efficient globin depletion, increases the number of detected transcripts. This amount of starting RNA also allows generating libraries of at least 5 nM, which is adequate for sequencing on any Illumina platform, even using a service provider. As the percentage of remaining rRNA and globin has a direct impact on the sequencing depth, we recommend 50 million paired-end reads per sample, which would be sufficient to fully characterize the transcripts, assuming good depletion (< 10%). As genomic DNA may also be isolated during RNA extraction, we recommend performing two rounds of DNase I treatment on RNA. In the absence of DNA, less than 10% of reads align to the undesirable strand or to the intergenic regions, a plateau is reached on the rarefaction curves, and there is low between-sample variability in the number of genes identified. Finally, to ensure the unique origin of RNA, at least 80% of the reads should align to the desired species genome, and a single GC content peak should be observed. If, for any reason, these recommendations cannot be achieved, we suggest reviewing that given stage of the workflow before processing any additional samples.

**Table 2:** Proposed guidelines for both wet- and dry-lab criteria. List of the important criteria at each step of the workflow and the corresponding recommended thresholds based on our study

Step	Criteria	Recommendation			
Extraction	RNA quantity	100 ng			
Library preparation	RNA molarity	> 5 nM			
Sequencing preprocessing	Sequencing depth	> 50 M reads			
	GC content	Unique peak			
Mapping	rRNA after depletion	< 10%			
	Globin after depletion	< 10%			
	Library strandness (% undesirable sens)	< 10%			
	Genome mapping	> 80%			
	Protein coding content	~ 75%			
Quantification	Rarefaction curves	Reach a plateau with homogeneity			
	Number of detected genes	Human: [12500-14000]	NHP: [12500–14000]	Mouse: [10500–12000]	Rabbit: [10500–12000]

Based on the performances of the proposed total RNA-Seq workflow, it can be suitable for the analysis of other types of samples such as swabs, saliva, biopsies or urine for transcriptomics or metatranscriptomics analysis. However, the extraction step and, in particular the conditions of lysis, would need to be adapted according to the starting matrix. Minor protocol modifications are expected for the subsequent steps, including library preparation and sequencing.

Our single transcriptomics workflow is compatible with automation on all major platforms, including Hamilton, Tecan, Biomek, and Eppendorf, supporting its generalized implementation. This includes the RNA extraction and the library preparation. Our extraction protocol is already integrated on the Maxwell RSC Instrument (Promega).

## Conclusions

We recommend the following workflow for blood transcriptomics analysis: (i) the use of PAXgene tubes for stabilizing RNA, with the buffer volume adapted in function of the volume of collected blood; (ii) RNA isolation using the Maxwell HT simplyRNA kit, Custom; (iii) Double DNase I treatment of RNA samples; (iv) RNA library preparation using the Zymo-Seq RiboFree Total RNA Library Kit; (v) sequencing with at least 50 million paired-end reads per sample; and (vi) quality controls of generated data using the RNASEQ-QC pipeline.

Although this total RNA-Seq workflow has only been tested on human, NHP, mouse, and rabbit samples, it is expected to work as well with other species. However, the requirement of additional technical setup cannot be excluded. We consider that such end-to-end workflow will help to streamline and standardize blood transcriptomics analyses in translational studies, including preclinical and clinical assessments. This, in turn, could help identify potential biomarkers for predicting the characteristics of an immune response, the reactogenicity of a vaccine, or the efficacy of a drug.

### Supplementary Information

**Supplementary Material 1.**

## Data Availability

The preclinical datasets generated during the current study are available on the BioProject database (ID PRJNA989407, https://www.ncbi.nlm.nih.gov/sra/PRJNA989407). The RNASEQ-QC pipeline is available on github (https://github.com/hhageBA/rnaseq-qc).

## Abbreviations

bp: Base pair
CNRS: Centre national de la recherche scientifique
DNA: Desoxyribonucleic acid
EFS: Etablissement français du sang
lncRNA: Long non-coding RNA
Misc_RNA: Miscellaneous RNA
mRNA: Messenger RNA
Mt_rRNA: Mitochondrial rRNA
NCBI: National center for biotechnology information
NGS: Next-Generation Sequencing
NHP: Non-Human primate
PCR: Polymerase chain reaction
Poly[A]: Polyadenylated RNA
QC: Quality control
RIN: RNA integrity number
RLE: Relative log expression
RNA: Ribonucleic acid
RNA-Seq: RNA-Sequencing
rRNA: Ribosomal RNA
TSS/TES: Transcription start site/end sites
