# Supplementary material for: A single workflow for multi-species blood transcriptomics
Source: BMC Genomics. 2024 Mar 16;25:282. doi: 10.1186/s12864-024-10208-2 (PMC10944614; doi:10.1186/s12864-024-10208-2)
Supplement: Supplementary file 1 — Supplementary Material 1. [file 12864_2024_10208_MOESM1_ESM.docx]

**Supplementary figures**


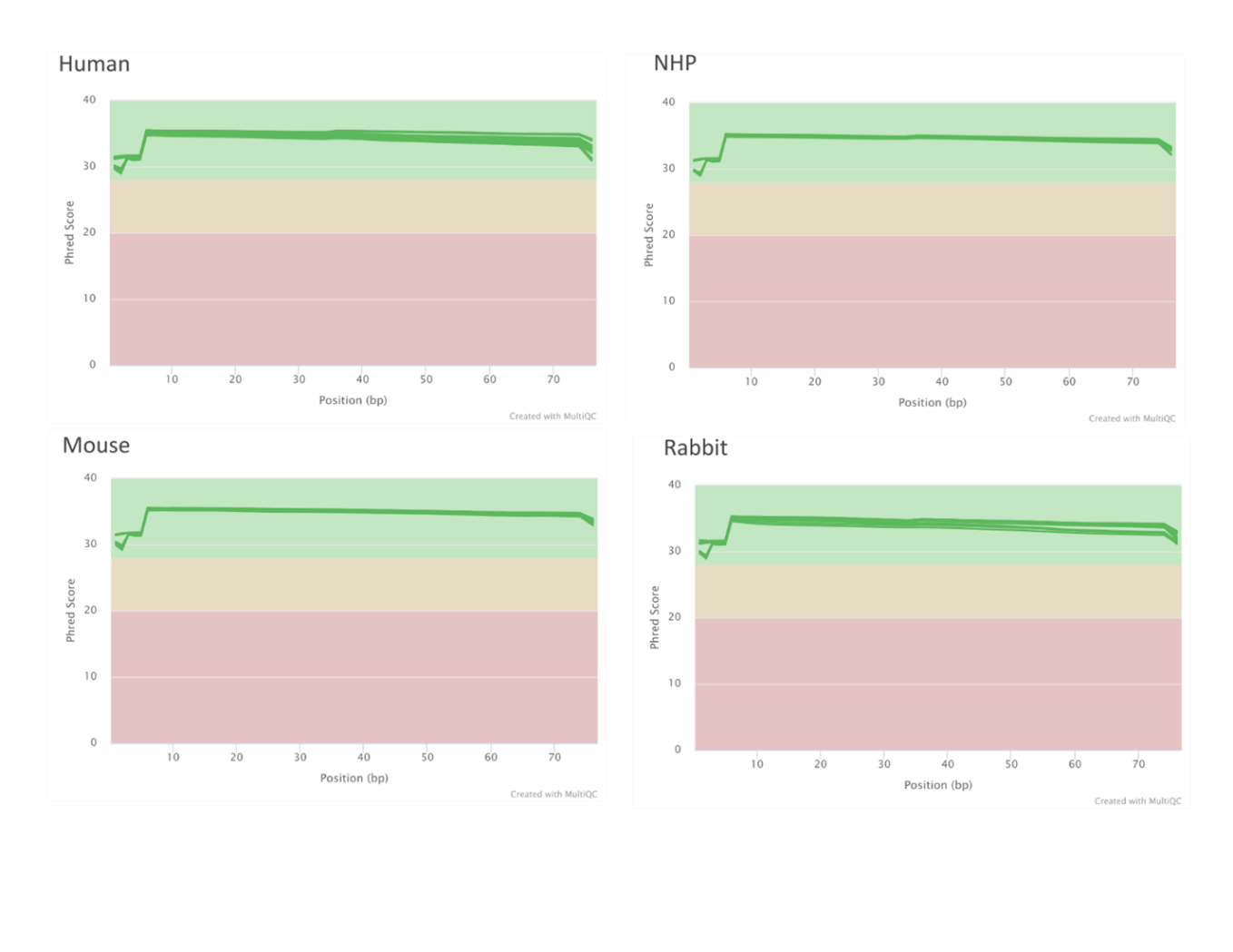


Supplementary Figure 1. Transcriptomics sequencing quality. Phred score at each position along the read for each species: human, NHP, mouse and rabbit. Each curve corresponds to one single sample.


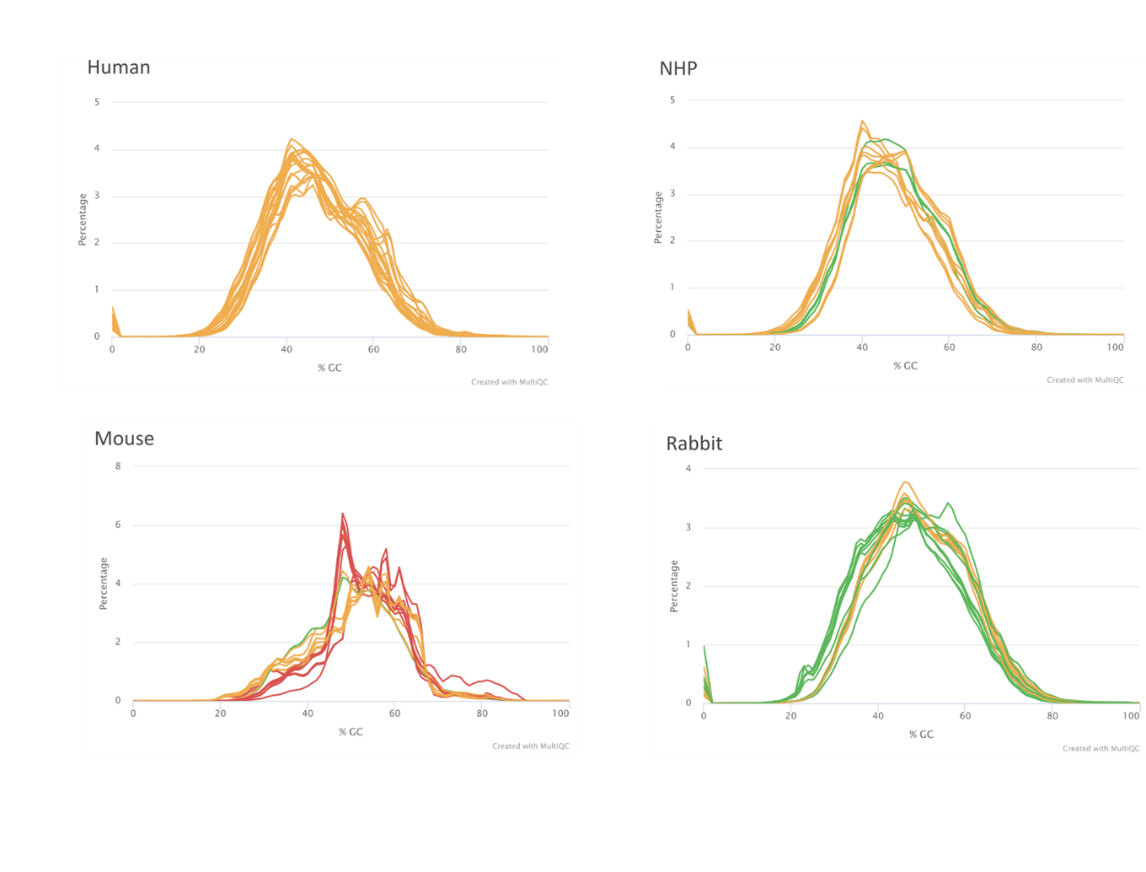


Supplementary Figure 2. GC content distribution. Percentage of GC content over the sequencing reads for each species: human, NHP, mouse and rabbit. Each curve corresponds to one single sample. Green curves indicate the presence of a single GC peak. Yellow curves suggest that of most of the reads have the same GC content, with few exceptions. Red curves highlight the presence of multiple GC peaks.


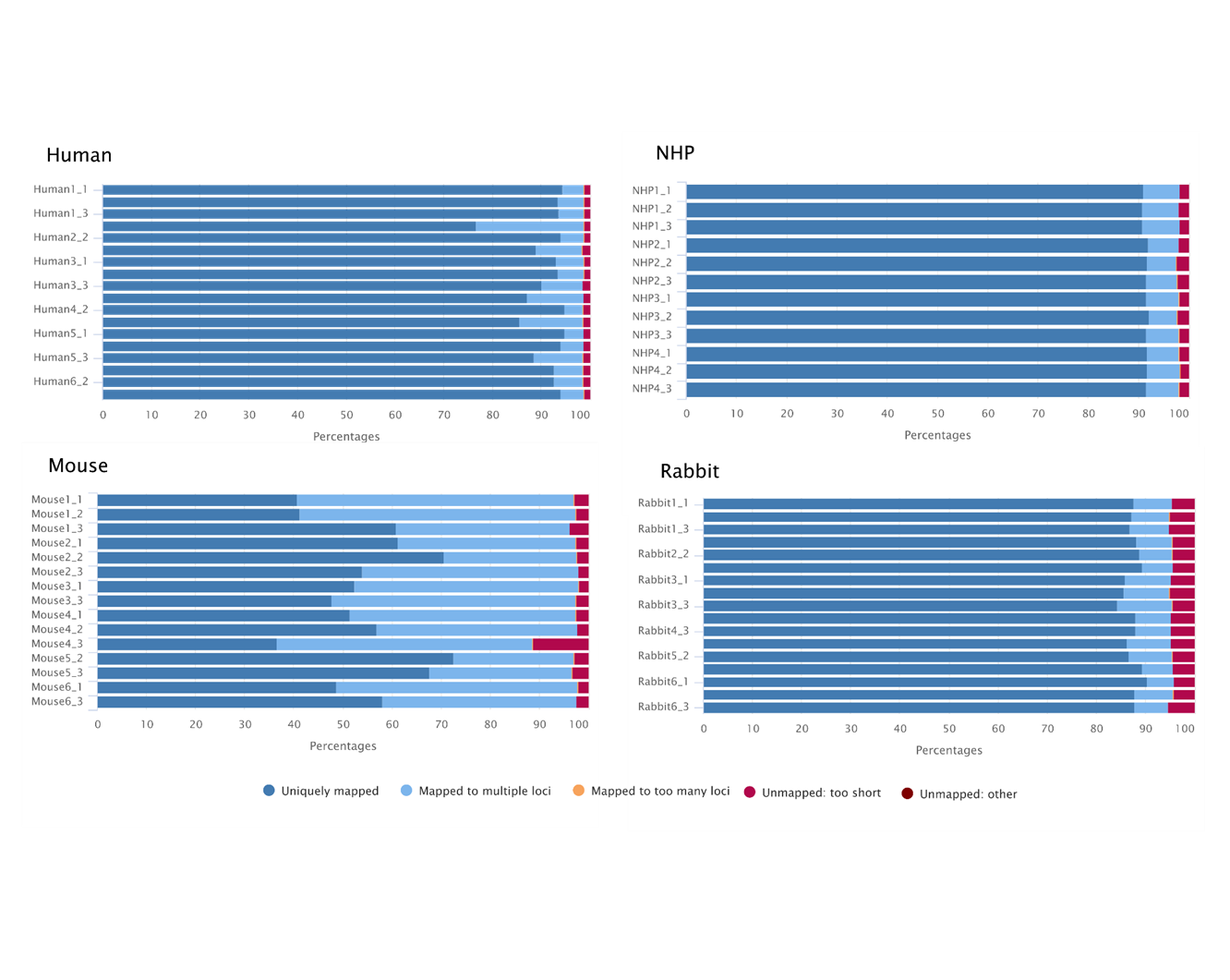


Supplementary Figure 3. Genome mapping. Percentage of reads mapping to the reference genome for each species: human, NHP, mouse and rabbit. Reads are classified in five categories: (dark blue) uniquely mapped to a single location in the genome; (light blue) mapped to multiple loci (more than one but less than 10 loci); (orange) mapped to too many loci (>10 loci); (Burgundy) unmapped: too short if the minimal required bp match is not satisfied (see Methods) and (brown) unmapped: other.


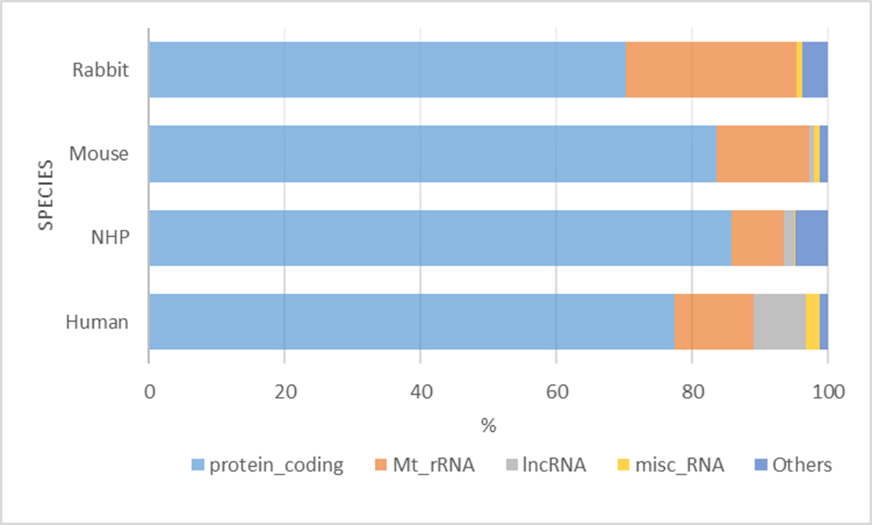


Supplementary Figure 4. Gene biotype. Mean percentage of reads mapping to different biotypes for all samples of each species: rabbit, mouse, NHP and human. The different biotypes are protein_coding (light blue), mitochondrial rRNA (Mt_rRNA, orange), long non-conding RNA (lncRNA, grey), miscellanous_RNA (misc_RNA, yellow) and others (dark blue).


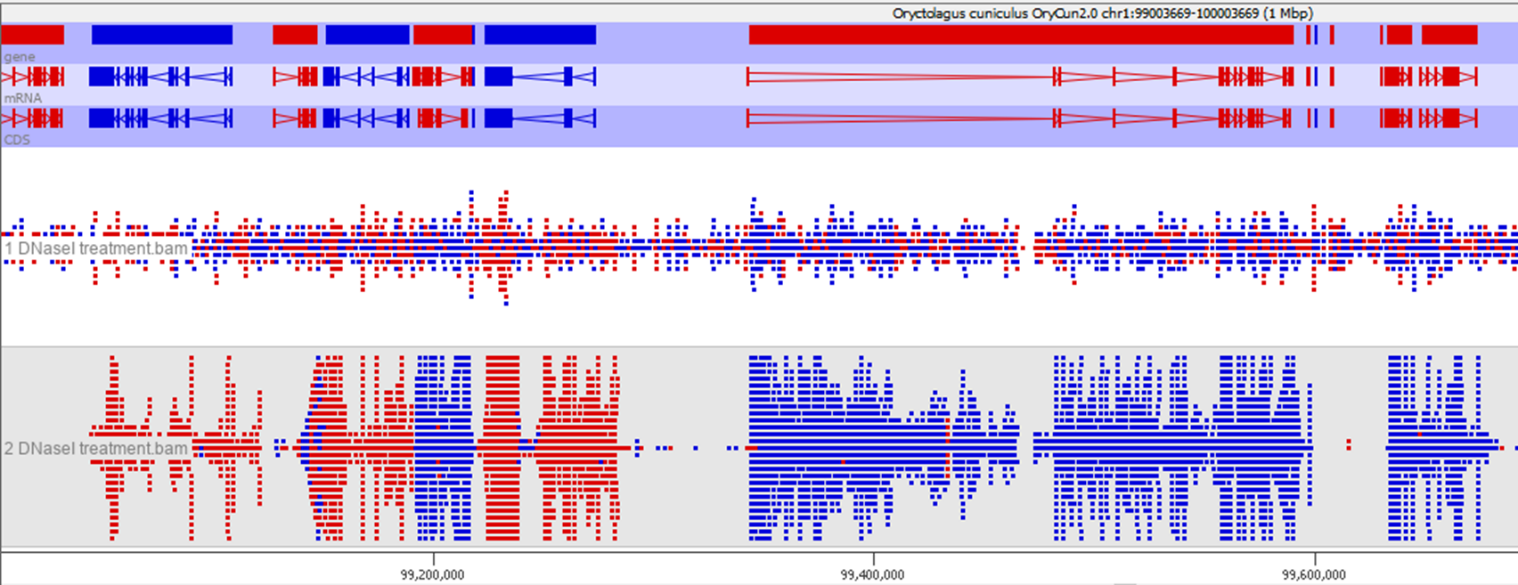


Supplementary Figure 5. Detection of residual genomic DNA in one rabbit sample. Mapping of the sequencing reads over the chromosome 1 of the *Oryctolagus cuniculus* genome (1 Mbp window; upper panel) in two conditions: (middle panel) RNA sample submitted to a single DNase I treatment and (lower panel) RNA sample submitted to double DNase I treatments. This figure is a screenshot from the SeqMonk visualization tool.

*
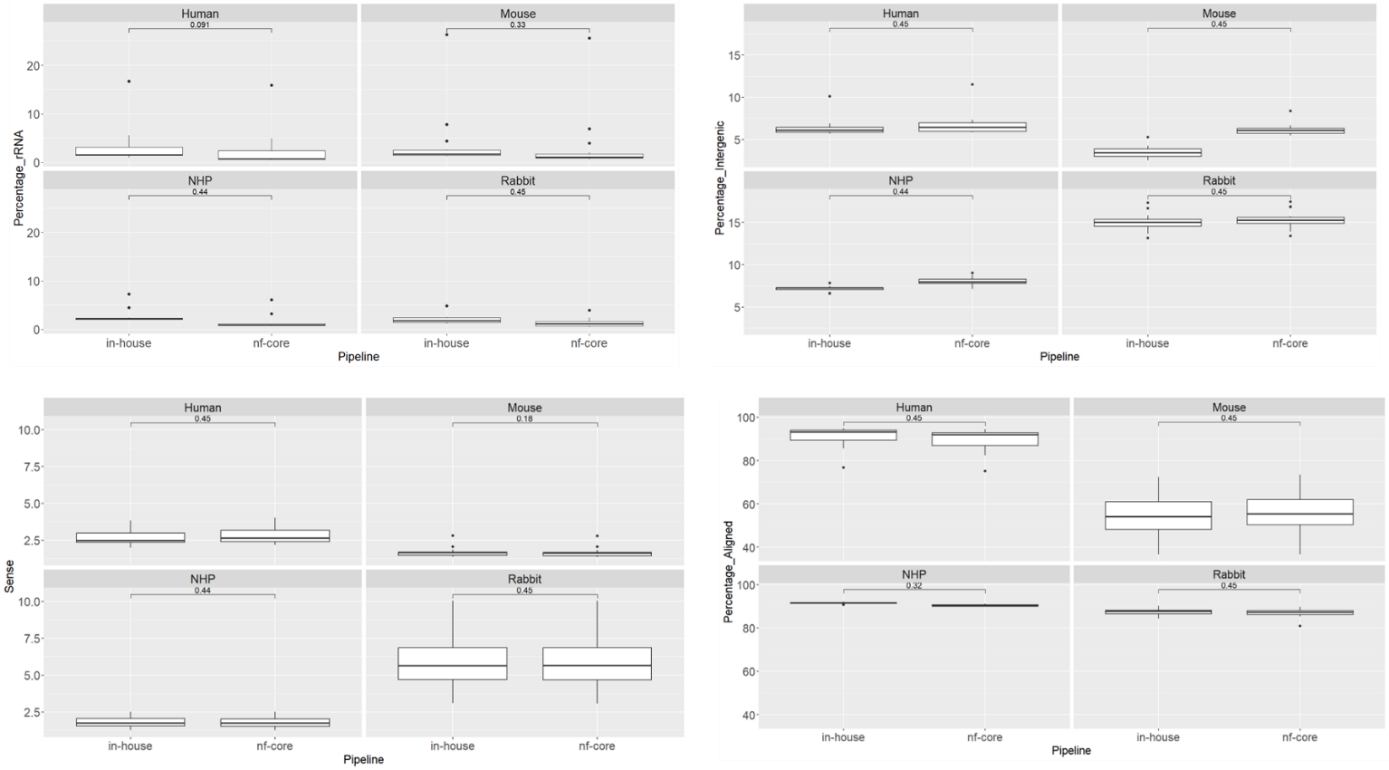
*

*
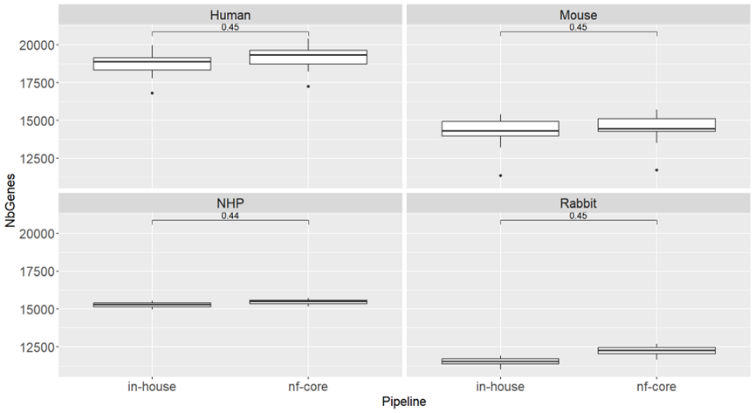
*

Supplementary Figure 6. Comparison of our RNASEQ-QC and nf-core pipelines for the analysis of transcriptomics data. The percentage of remaining rRNA after depletion, percentage of sense reads, percentage of intergenic reads, percentage of aligned reads and the number of expressed genes detected were compared between the two bioinformatic pipelines for Human, NHP, Mouse and Rabbit samples.


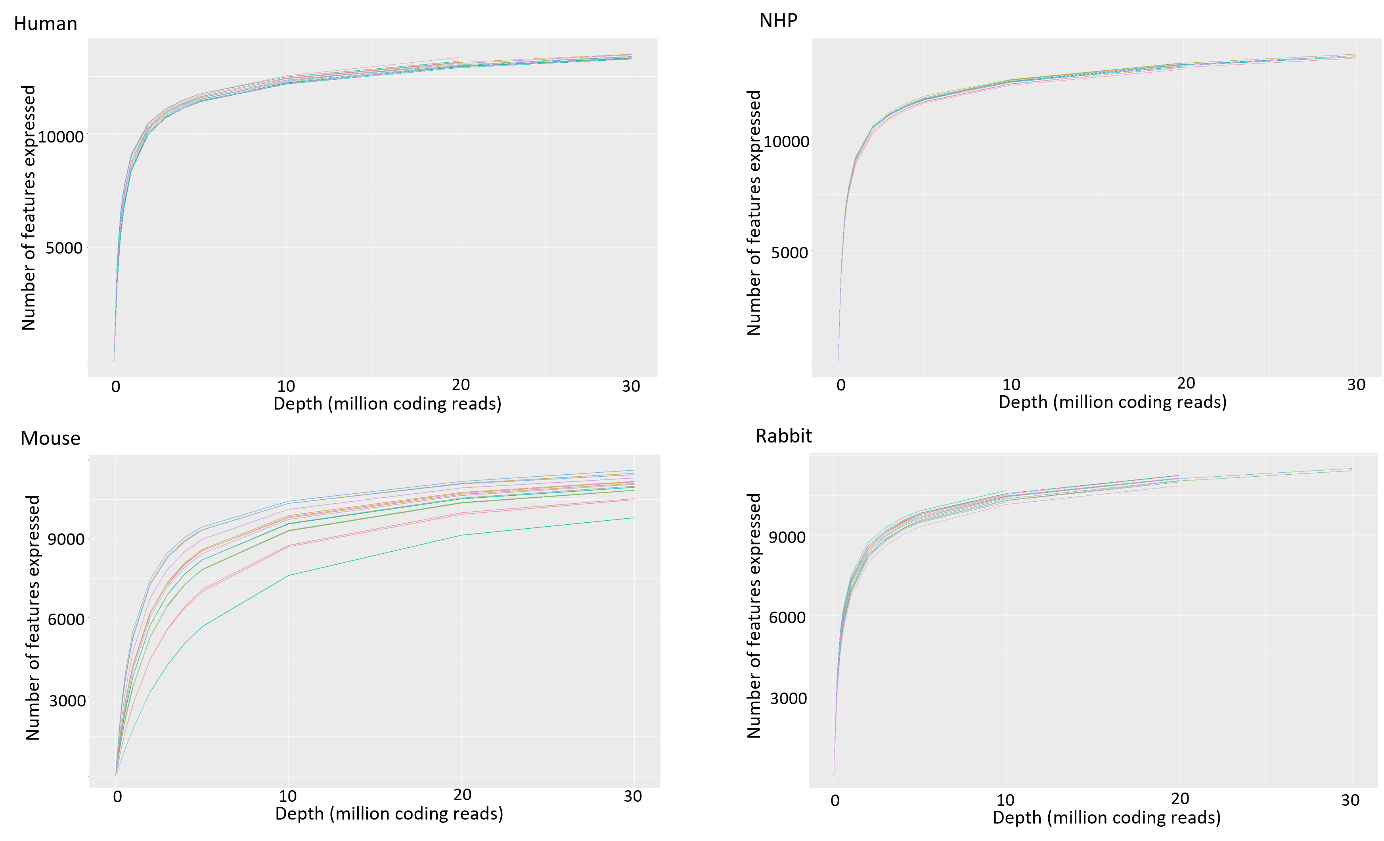


Supplementary Figure 7. Rarefaction curves. Number of expressed features compared to the sequencing depth for Human, NHP, Mouse and Rabbit samples. Each curve in each plot represents a sample.

**Supplementary Tables**

Supplementary Table1. Quantity and quality of the extracted RNA samples. RNA concentration (ng/µL) and quality (RIN) were measured by migration of RNA on the Fragment Analyzer or Bioanalyzer. N/A: Not available. The quantity (µg) was assessed by multiplying the concentration (ng/µL) by the elution volume (15 µL).

| **ID** | **Concentration (ng/µL)** | **Quantity (µg)** | **RIN** |
| --- | --- | --- | --- |
| Human1_1 | 191.6 | 2.87 | 8.4 |
| Human1_2 | 202.3 | 3.03 | 8.5 |
| Human1_3 | 207.4 | 3.11 | 8.7 |
| Human2_1 | 443.3 | 6.65 | 9.1 |
| Human2_2 | 128.4 | 1.93 | 9.1 |
| Human2_3 | 310 | 4.65 | 8.9 |
| Human3_1 | 138.1 | 2.07 | 8.8 |
| Human3_2 | 179.4 | 2.69 | 9 |
| Human3_3 | 227.5 | 3.41 | 8.7 |
| Human4_1 | 612.2 | 9.18 | 9 |
| Human4_2 | 19.5 | 0.29 | 8.9 |
| Human4_3 | 354.1 | 5.31 | 8.6 |
| Human5_1 | 77 | 1.16 | 9.3 |
| Human5_2 | 180.2 | 2.7 | 9 |
| Human5_3 | 185.1 | 2.78 | 9.1 |
| Human6_1 | 113 | 1.69 | 8.6 |
| Human6_2 | 238.7 | 3.58 | 8.2 |
| Human6_3 | 514.7 | 7.72 | 8.4 |
| NHP1_1 | 17.8 | 0.27 | 9.9 |
| NHP1_2 | 27.5 | 0.41 | 10 |
| NHP1_3 | 20.9 | 0.31 | 10 |
| NHP2_1 | 11.4 | 0.17 | 9.8 |
| NHP2_2 | N/A | N/A | 10 |
| NHP2_3 | 13.6 | 0.2 | 10 |
| NHP3_1 | 16 | 0.24 | 10 |
| NHP3_2 | 14.9 | 0.22 | 10 |
| NHP3_3 | 29.1 | 0.44 | 9.9 |
| NHP4_1 | 47.4 | 0.71 | 9.9 |
| NHP4_2 | 25 | 0.38 | 10 |
| NHP4_3 | 36.1 | 0.54 | 9.8 |
| Mouse1 _1 | 13.4 | 0.13 | 8.7 |
| Mouse1 _2 | 17.9 | 0.18 | 9.2 |
| Mouse1 _3 | 19.6 | 0.2 | 8.9 |
| Mouse2 _1 | 20.16 | 0.2 | 9 |
| Mouse2 _2 | 11.89 | 0.12 | 9.1 |
| Mouse2 _3 | 10.9 | 0.11 | 8.9 |
| Mouse3 _1 | 7.26 | 0.07 | 9 |
| Mouse3 _2 | 2.5 | 0.03 | N/A |
| Mouse3 _3 | 9.8 | 0.1 | 9.1 |
| Mouse4 _1 | 15 | 0.15 | 9 |
| Mouse4 _2 | 6.55 | 0.07 | 9.2 |
| Mouse4 _3 | 11.5 | 0.12 | 9.1 |
| Mouse5 _1 | 4.2 | 0.04 | 9.1 |
| Mouse5 _2 | 31.4 | 0.31 | 9 |
| Mouse5 _3 | 19.3 | 0.19 | 9.2 |
| Mouse6 _1 | 7.3 | 0.07 | 8.8 |
| Mouse6 _2 | 2.03 | 0.02 | N/A |
| Mouse6 _3 | 14.4 | 0.14 | 9.2 |
| Rabbit1_1 | 74 | 1.11 | 9.5 |
| Rabbit1_2 | 77.9 | 1.17 | 9.6 |
| Rabbit1_3 | 109 | 1.64 | 9.5 |
| Rabbit2_1 | 74 | 1.11 | 9.6 |
| Rabbit2_2 | 130 | 1.94 | 9.7 |
| Rabbit2_3 | 111 | 1.66 | 9.6 |
| Rabbit3_1 | 125 | 1.87 | 9.4 |
| Rabbit3_2 | 116 | 1.74 | 9.6 |
| Rabbit3_3 | 238 | 3.56 | 9.3 |
| Rabbit4_1 | 96 | 1.43 | 9.8 |
| Rabbit4_3 | 64 | 0.96 | 9.5 |
| Rabbit5_1 | 100.2 | 1.5 | 9.5 |
| Rabbit5_2 | 87.7 | 1.32 | 9.5 |
| Rabbit5_3 | 86.4 | 1.3 | 10 |
| Rabbit6_1 | 61.3 | 0.92 | 9.9 |
| Rabbit6_2 | 99.2 | 1.49 | 9.7 |
| Rabbit6_3 | 153.5 | 2.3 | 10 |

Supplementary Table2. Quality controls of the sequencing libraries with the corresponding number of raw read pairs generated. The size of the library fragments was estimated following Fragment Analyzer migration. The molarity was calculated from the concentration measured by fluorescence and the fragment size. The raw read pairs was assessed following sequencing demultiplexing.

| **ID**​ | **Molarity (nM)**​ | **Average Size (bp)**​ | **Raw read pairs (M)**​ |
| --- | --- | --- | --- |
| Human1_1​ | 156.6​ | 397​ | 96​ |
| Human1_2​ | 126.8 | 385​ | 77​ |
| Human1_3​ | 165.3 | 373​ | 116​ |
| Human2_1​ | 48.2 | 378​ | 147​ |
| Human2_2​ | 181.3 | 349​ | 116​ |
| Human2_3​ | 19.7 | 378​ | 154​ |
| Human3_1​ | 122.8 | 373​ | 79​ |
| Human3_2​ | 115.7 | 389​ | 122​ |
| Human3_3​ | 74.4 | 405​ | 131​ |
| Human4_1​ | 69.1 | 383​ | 121​ |
| Human4_2​ | 195.7 | 363​ | 88​ |
| Human4_3​ | 69.9 | 379​ | 138​ |
| Human5_1​ | 225 | 373​ | 124​ |
| Human5_2​ | 162.3 | 383​ | 145​ |
| Human5_3​ | 90.4 | 403​ | 106​ |
| Human6_1​ | 190.8 | 331​ | 158​ |
| Human6_2​ | 182.4 | 377​ | 144​ |
| Human6_3​ | 29.3 | 378​ | 201​ |
| NHP1_1​ | 243 | 395​ | 119​ |
| NHP1_2​ | 203.4 | 402​ | 119​ |
| NHP1_3​ | 229 | 389​ | 141​ |
| NHP2_1​ | 296.8 | 368​ | 105​ |
| NHP2_2​ | 142.9 | 398​ | 104​ |
| NHP2_3​ | 203.5 | 408​ | 130​ |
| NHP3_1​ | 213.2 | 404​ | 127​ |
| NHP3_2​ | 100.3 | 406​ | 133​ |
| NHP3_3​ | 204.1 | 376​ | 131​ |
| NHP4_1​ | 164.2 | 382​ | 123​ |
| NHP4_2​ | 187.9 | 374​ | 128​ |
| NHP4_3​ | 109.3 | 375​ | 139​ |
| Mouse1 _1 ​ | 58.2 | 428​ | 106​ |
| Mouse1 _2 ​ | 42.9 | 432​ | 114​ |
| Mouse1 _3 ​ | 37.8 | 454​ | 127​ |
| Mouse2 _1 ​ | 33.2 | 439​ | 120​ |
| Mouse2 _2 ​ | 23.5 | 444​ | 104​ |
| Mouse2 _3 ​ | 22.6 | 440​ | 108​ |
| Mouse3 _1 ​ | 50.9 | 426​ | 102​ |
| Mouse3 _3 ​ | 22.7 | 428​ | 115​ |
| Mouse4 _1 ​ | 22.9 | 415​ | 106​ |
| Mouse4 _2 ​ | 21.2 | 430​ | 98​ |
| Mouse4 _3 ​ | 19.4 | 412​ | 91​ |
| Mouse5 _2 ​ | 14 | 467​ | 117​ |
| Mouse5 _3 ​ | 32.8​ | 455​ | 129​ |
| Mouse6 _1 ​ | 28.4 | 460​ | 97​ |
| Mouse6 _3 ​ | 29.1 | 450​ | 118​ |
| Rabbit1_1​ | 124.6 | 419​ | 107​ |
| Rabbit1_2​ | 131.1 | 424​ | 121​ |
| Rabbit1_3​ | 176.3 | 405​ | 115​ |
| Rabbit2_1​ | 173 | 409​ | 134​ |
| Rabbit2_2​ | 21.3 | 452​ | 127​ |
| Rabbit2_3​ | 133.2 | 398​ | 144​ |
| Rabbit3_1​ | 63 | 394​ | 154​ |
| Rabbit3_2​ | 107.2 | 403​ | 140​ |
| Rabbit3_3​ | 37.5 | 383​ | 81​ |
| Rabbit4_1​ | 112.7 | 395​ | 82​ |
| Rabbit4_3​ | 113.5 | 393​ | 140​ |
| Rabbit5_1​ | 102.5 | 403​ | 118​ |
| Rabbit5_2​ | 113.3 | 377​ | 90​ |
| Rabbit5_3​ | 134.9 | 371​ | 164​ |
| Rabbit6_1​ | 242.4 | 313​ | 165​ |
| Rabbit6_2​ | 67.2 | 380​ | 106​ |
| Rabbit6_3​ | 93 | 392​ | 94​ |
